# Metabolic and Cardiac Adaptation to Chronic Pharmacologic Blockade of Facilitative Glucose Transport in Murine Dilated Cardiomyopathy and Myocardial Ischemia

**DOI:** 10.1038/s41598-018-24867-1

**Published:** 2018-04-24

**Authors:** Monique R. Heitmeier, Maria A. Payne, Carla Weinheimer, Attila Kovacs, Richard C. Hresko, Patrick Y. Jay, Paul W. Hruz

**Affiliations:** 1 Department of Pediatrics, Washington University School of Medicine, St. Louis, USA; 2 Department of Internal Medicine, Washington University School of Medicine, St. Louis, USA; 3 Department of Genetics, Washington University School of Medicine, St. Louis, USA; 4 Department of Cell Biology and Physiology, Washington University School of Medicine, St. Louis, USA

## Abstract

GLUT transgenic and knockout mice have provided valuable insight into the role of facilitative glucose transporters (GLUTs) in cardiovascular and metabolic disease, but compensatory physiological changes can hinder interpretation of these models. To determine whether adaptations occur in response to GLUT inhibition in the failing adult heart, we chronically treated TG9 mice, a transgenic model of dilated cardiomyopathy and heart failure, with the GLUT inhibitor ritonavir. Glucose tolerance was significantly improved with chronic treatment and correlated with decreased adipose tissue retinol binding protein 4 (RBP4) and resistin. A modest improvement in lifespan was associated with decreased cardiomyocyte brain natriuretic peptide **(**BNP) expression, a marker of heart failure severity. GLUT1 and −12 protein expression was significantly increased in left ventricular (LV) myocardium in ritonavir-treated animals. Supporting a switch from fatty acid to glucose utilization in these tissues, fatty acid transporter CD36 and fatty acid transcriptional regulator peroxisome proliferator-activated receptor α (PPARα) mRNA were also decreased in LV and soleus muscle. Chronic ritonavir also increased cardiac output and dV/dt-d in C57Bl/6 mice following ischemia-reperfusion injury. Taken together, these data demonstrate compensatory metabolic adaptation in response to chronic GLUT blockade as a means to evade deleterious changes in the failing heart.

## Introduction

The healthy heart hydrolyzes ~0.5 µmol/g wet weight per second of ATP for normal contractile function^1^. Greater than 70% of this ATP is generated from the oxidation of fatty acids (FA) and, to a lesser extent, utilization of other substrates such as carbohydrates and amino acids. In the stressed or failing heart, FA as a fuel source decreases and glucose, via increased glycolysis becomes a primary source of ATP production in the myocardium. Many patients with heart failure also suffer from insulin resistance, which further exacerbates myocardial dysfunction^2^. While it has been postulated that heart failure may lead to insulin resistance resulting in further decrease in cardiac function^3^, and insulin resistance is detrimental to cardiac outcomes in patients^4^, the effects of altered glucose homeostasis on heart failure progression remains to be elucidated.

Several genetic models have been generated in an effort to determine the role of glucose homeostasis and metabolism on cardiac function. Glucose is transported by a family of facilitative hexose transporters known as GLUTs^5^. Of the 14 known members, the ubiquitously expressed GLUT1 and insulin-responsive GLUT4 are the primary glucose transporters in the heart. Mice expressing GLUT1 under the α-myosin heavy chain promoter are protected from pressure overload-induced heart failure^6^ but not high fat diet-induced cardiac dysfunction^7^. The latter is due to a failure to upregulate fatty acid oxidation in the heart and the subsequent increased cardiac fatty acid load results in oxidative stress. Whole body or cardiac-specific GLUT4 ablation leads to cardiac hypertrophy and heart failure associated with reduced fatty-acid oxidation in the heart and hyperinsulinemia^8,9^. GLUT8, and −12 protein expression is significantly increased in left ventricle of GLUT4 knockout mice^10^, and a ~4-fold increase in the expression of GLUT12 has been observed in the left ventricle of the pacing-induced canine model of cardiac hypertrophy^11^. These results implicate additional GLUTs in myocardial glucose transport. Like GLUT4, GLUT12 is insulin-responsive and transgenic mice overexpressing GLUT12 have improved systemic glucose tolerance and insulin sensitivity^12^. These data suggest that additional signals or expression of other GLUT isoforms may preserve cardiac function and have metabolic benefit. While these genetic models have provided key insights into mechanisms associated with cardiac dysfunction as a result of impaired glucose homeostasis, compensatory mechanisms may exist as the modifications are generally present at birth. Therefore, pharmacologic disruption of facilitative glucose transport provides an alternate means to investigate myocardial effects with the advantage that the timing, duration and degree of blockade can be more readily modulated.

We have extensively examined the effects of glucose transport inhibitors on whole-body glucose homeostasis and functional effects in insulin-responsive tissues. Specifically, we have identified HIV protease inhibitors (PIs) as antagonists of GLUT function through direct and reversible binding to the transporter^13,14^. As these drugs require access to the glucose binding site from the cytosolic side of the protein, they act as non-competitive inhibitors of glucose import^15,16^. Several PIs including indinavir have been shown to be selective for GLUT4 over GLUT1. Others like ritonavir target both GLUT1 and GLUT4. PIs have been an integral component of combined antiretroviral treatment (cART) regimens where they have contributed significantly to the remarkable reduction in HIV-associated morbidity and mortality achieved over the past two decades^17^. As expected, GLUT blockade acutely (i.e. within minutes) induces systemic insulin resistance with impaired glucose tolerance. Importantly, this acute effect is reversible with drug removal^18^. With chronic drug exposure, visceral adiposity, hyperlipidemia, and insulin resistance refractory to drug withdrawal are observed^19^. Each of these effects are known to contribute to the development of cardiovascular disease^20^. Over the last decade, several basic science and clinical studies have contributed to the elucidation of the molecular mechanisms that lead to PI-induced insulin resistance^13,21–23^. Since the heart, like skeletal muscle, is an insulin responsive tissue, it has been postulated that some of the adverse cardiac effects of PI use may be due to direct effects of glucose transport blockade.

To determine the effects of sustained glucose blockade on the development of HF in the context of peripheral insulin resistance and glucose intolerance, we treated TG9 mice, a rodent model of dilated cardiomyopathy that progresses to heart failure and death within a predictable time frame, with ritonavir to pharmacologically inhibit GLUTs 1 and 4. We have previously shown that acute exposure of TG9 mice to ritonavir exacerbates heart failure and significantly decreases lifespan^24,25^. These effects are associated with decreased glucose intolerance and increased peripheral insulin resistance due to decreased glucose uptake in skeletal muscle and heart. To investigate the effects of chronic GLUT blockade, ritonavir was serially administered to TG9 mice prior to the development of insulin resistance, glucose intolerance and dilated cardiomyopathy. We also chronically administered ritonavir to C57BL/6 mice prior to myocardial ischemia to determine the effects of GLUT inhibition on cardiac function in the absence of insulin resistance and glucose intolerance. We report here the effects of chronic inhibition of GLUTs −1 and 4- in TG9 mice on glucose homeostasis, myocardial function and survival with characterization of changes in GLUT expression and mediators of insulin sensitivity. Chronic GLUT inhibition also did not further exacerbate the deleterious effects of ischemia reperfusion in C57BL/6 mice, but instead modestly improved cardiac output. Taken together, these data provide novel insight into compensatory mechanisms that abrogate perturbation of GLUT function. In addition to shedding light on potential effects in treated HIV infected patients, these data demonstrate the importance of incorporating homeostatic adaptations to targeted protein disruption in elucidating molecular mechanisms of disease pathogenesis.

## Results

**Figure 1:**
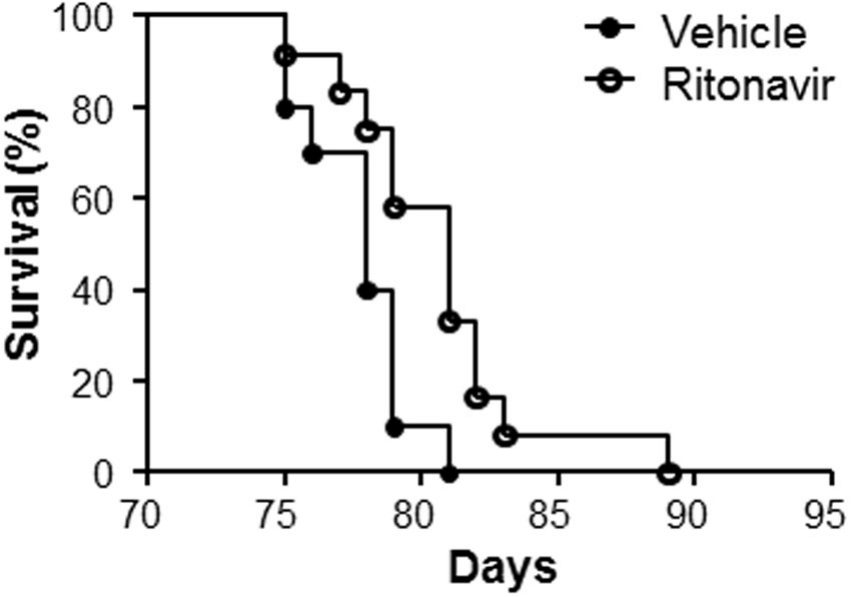
Chronic Ritonavir Treatment Prolongs Survival in TG9 Mice. Kaplan-Meier curve of female TG9 mice treated via intraperitoneal injection with ritonavir (10 mg/kg, n = 12) or vehicle (10% EtOH in normal saline, n = 10) daily starting at six weeks of age and continuing until time of death, p-value < 0.05.

**Figure 2:**
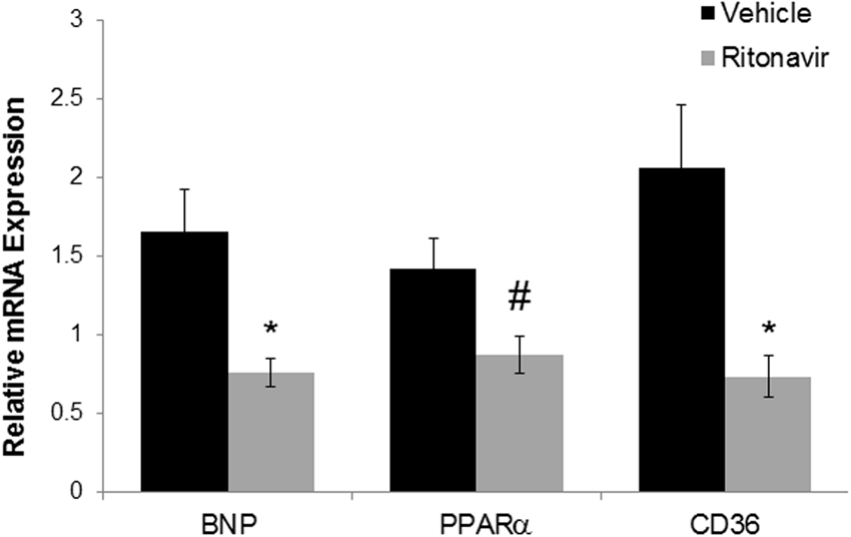
Effects of Chronic Ritonavir Treatment on BNP, PPARα and CD36 Expression by Left Ventricle. The relative mRNA expression of BNP, PPARα, and CD36 in LV of 75 day old male TG9 mice, treated with vehicle or ritonavir since 6 weeks of age, was examined by RT-PCR as described in Methods. (n = 4–5/group). *p < 0.01 vs. Veh, ^#^p < 0.05 vs. Veh.

The TG9 mouse, a murine model of dilated cardiomyopathy, has proven valuable in elucidating the role of glucose homeostasis on cardiac function^25^. This model was generated by the cardiac-specific overexpression of the Cre recombinase protein under the α-myosin promoter. TG9 mice develop progressive heart failure and predictably die within a narrow window between 11 and 13 weeks of life^26^. Captopril, an ACE inhibitor which improves heart failure survival in humans, significantly increases lifespan in these animals by 7–10 days^26^. We previously demonstrated that the acute blockade of GLUT4 by HIV protease inhibitors in 75 day old TG9 mice, which have signs of cardiomyopathy, causes abrupt, decompensated heart failure and death^24,25^. To determine the effects of chronic GLUT inhibition of glucose uptake on mortality, ritonavir was administered to TG9 mice for 4 weeks, starting at 6 weeks of age. In contrast to the mortality associated with acute administration, chronic ritonavir exposure resulted in a modest but significant increase in lifespan compared to vehicle-treated animals (80.6 ± 1.01 vs. 77.8 ± 0.61, p < 0.05, Fig. 1). mRNA levels of BNP, which correlates with heart failure severity, was decreased ~55% in TG9 mice treated chronically with ritonavir as compared to vehicle controls supporting the improvement in cardiac outcome for these mice (Fig. 2). Nuclear hormone receptor PPARα, and fatty acid receptor CD36 which play a critical role in FA metabolism in the heart, especially when the heart is stressed or in heart failure, were decreased in left ventricle of chronic ritonavir-treated animals by ~40% and ~64%, respectively (Fig. 2). These results demonstrate that improved survival in this animal model of heart failure is associated with decreased markers of fatty acid uptake and oxidation.

**Figure 3:**
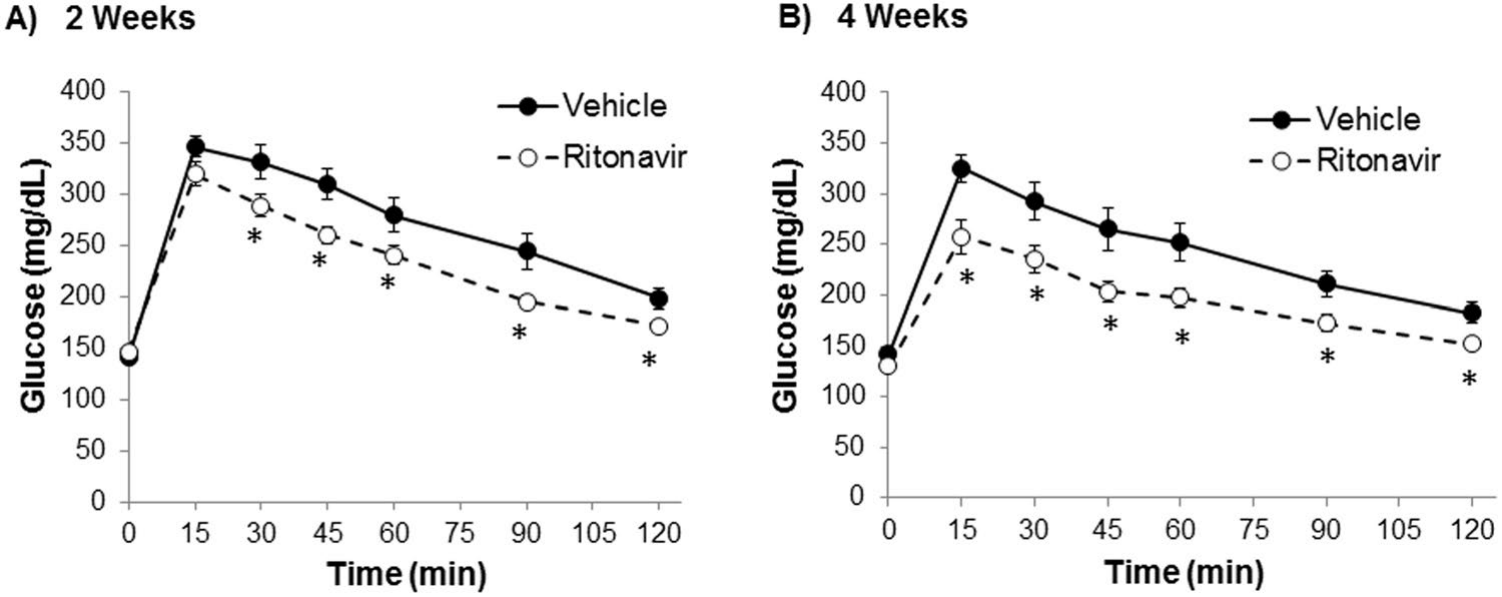
Chronic Ritonavir Improves Glucose Tolerance in TG9 Mice. Oral glucose tolerance test was performed on male TG9 mice at (**A**) 8 weeks of age (2 weeks treatment) and, (**B**) 10 weeks of age (4 weeks treatment) following a 5 hr fast and as described in Methods. n = 10–12/group. *p < 0.05 vs. Veh.

**Figure 4:**
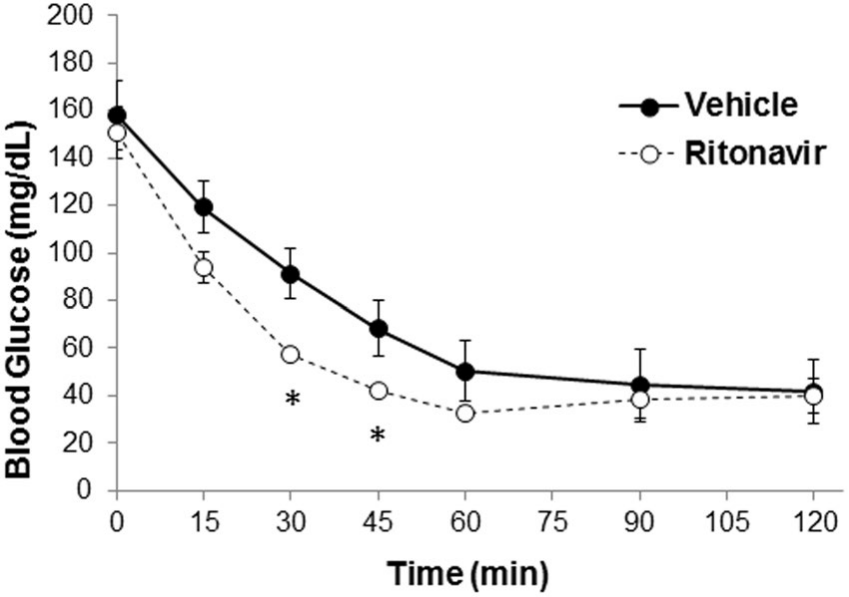
Effects of Chronic Ritonavir on Insulin Sensitivity in TG9 Mice. Insulin tolerance tests were performed following a 5 hr fast on male TG9 mice that had been treated with ritonavir or vehicle for 3 weeks as described in Methods. (n = 4–6/group). *p < 0.05 vs. Veh.

**Figure 5:**
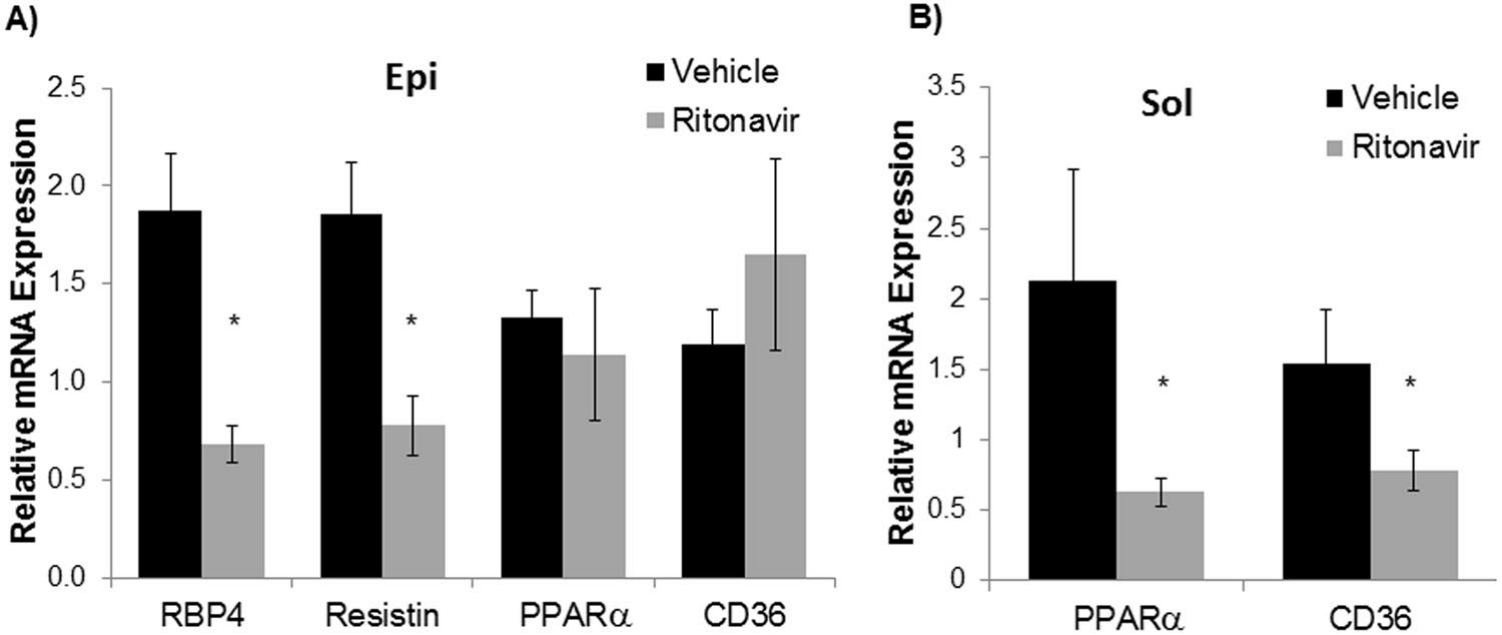
Effects of Chronic Ritonavir Treatment on Inflammation and Insulin Resistance-related Gene Expression by Adipose and Soleus Tissue. The relative mRNA expression of RBP4, resistin, PPARα and CD36 in (**A**) epididymal adipose, and PPARα and CD36 in (**B**) soleus muscle of 75 day old male TG9 mice, treated with vehicle or ritonavir since 6 weeks of age, was examined by RT-PCR as described in Methods. (n = 4–5/group). *p < 0.01 vs. Veh.

**Figure 6:**
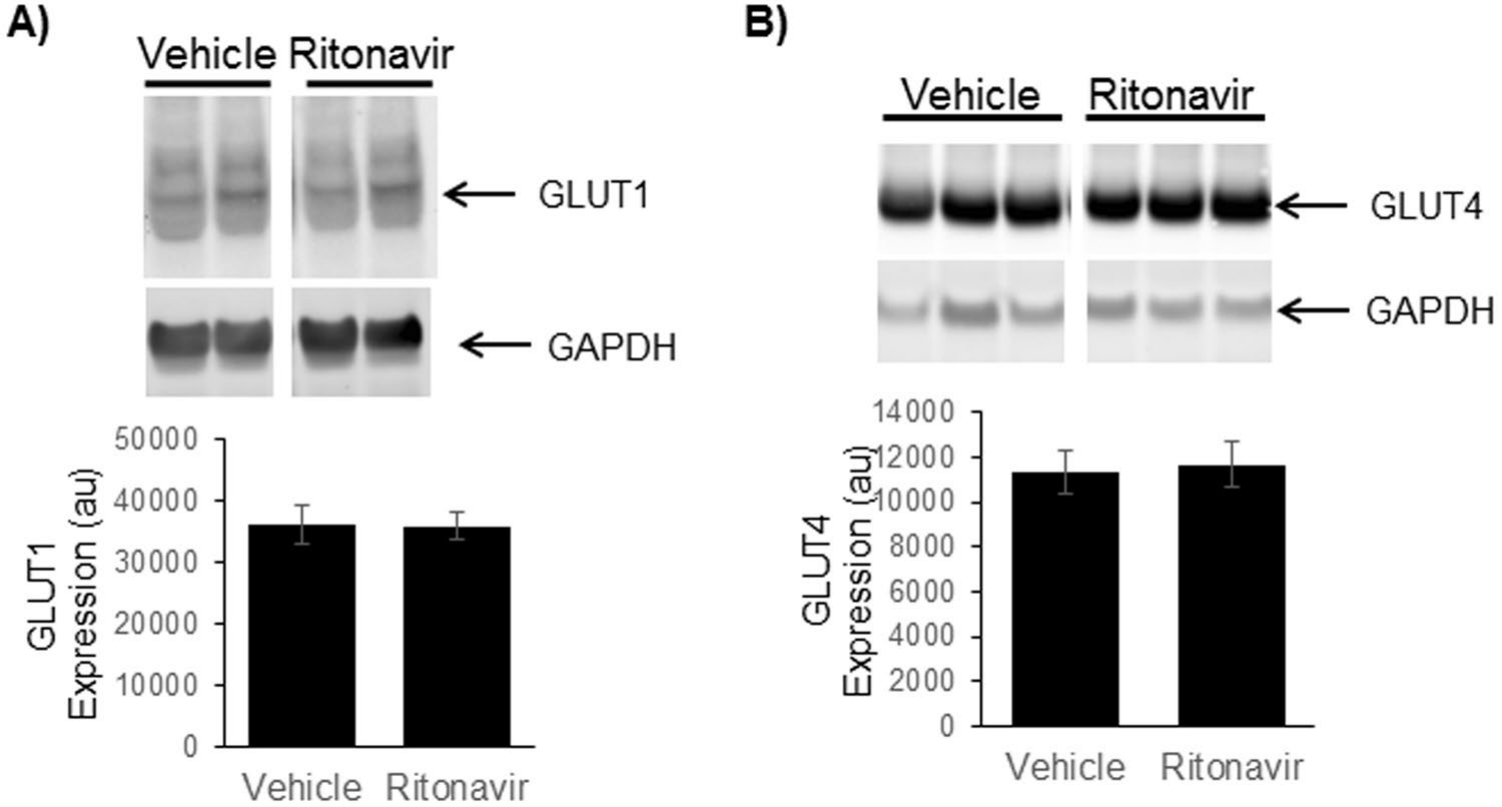
Effects of Chronic Ritonavir Treatment on GLUT Protein Expression by Gastrocnemius Muscle. Protein expression of (**A**) GLUT1 and (**B**) GLUT4 were determined in 75 day old male TG9 mice, treated with vehicle or ritonavir since 6 weeks of age, by Western Blot analysis as described in Methods. Western blots are representative data for n = 6 animals/group. Representative full-length blots are presented in Supplementary Fig. 1. Quantitative data are normalized to GAPDH controls and are shown as means ± SEM for n = 6 animals/group *p < 0.01 vs. Veh.

Ritonavir induces peripheral insulin resistance and reversible hyperglycemia *in vivo* when administered acutely^13,18^. Following 6 weeks of ritonavir exposure, glucose tolerance in C57Bl/6 J mice was no different than vehicle-treated animals (AUC 21552 ± 2000 vs. 23234 ± 1095, respectively). To determine the effects of chronic drug exposure on glucose tolerance and insulin sensitivity, oral glucose and insulin tolerance tests were performed in TG9 animals treated with either vehicle or ritonavir. Ritonavir-treated animals exhibited improved glucose tolerance after 2 weeks of treatment (AUC 32482 ± 1447 vs. 27984 ± 716, p < 0.05, Fig. 3A). The effect persisted through treatment duration (AUC 29048 ± 1332 vs. 23322 ± 965, p < 0.05 Fig. 3B). Insulin sensitivity was also improved in ritonavir-treated animals (AUC 8395 ± 1356 vs. 6519 ± 393, Fig. 4). The improvement in glucose tolerance and insulin sensitivity in TG9 mice correlated with a ~60% decrease in insulin resistance markers retinol-binding protein 4 (RBP4) and resistin in epidydimal fat pads isolated from ritonavir-treated animals (Fig. 5A). Although no difference in PPARα and CD36 mRNA levels were observed in epidydimal fat pads from treated and non-treated mice (Fig. 5A), a ~70% decrease in PPARα and ~50% decrease in CD36, respectively, was observed in soleus muscle isolated from ritonavir-treated animals vs. vehicle controls (Fig. 5B). As muscle is a primary glucose disposal site, contributing ~80% to glucose homeostasis, we determined whether improved glucose tolerance and insulin sensitivity correlated with changes in muscle GLUT expression. As shown in Fig. 6 GLUT1 and GLUT4 were similar between vehicle and ritonavir-treated animals.These results show that improved glucose homeostasis in ritonavir-treated animals is associated with decreased markers of insulin resistance and fatty acid oxidation.

**Figure 7:**
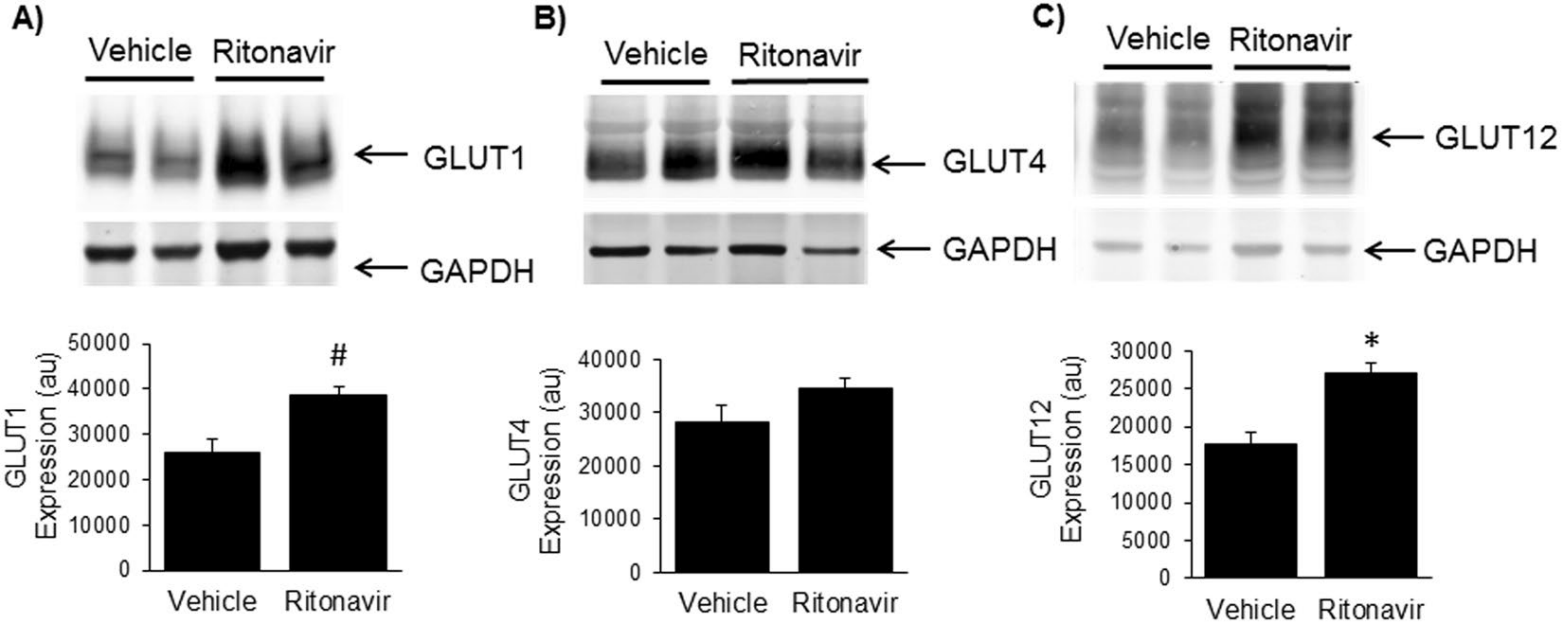
Effects of Chronic Ritonavir Treatment on GLUT Protein Expression by Left Ventricle. Protein expression of (**A**) GLUT1, (**B**) GLUT4, and (**C**) GLUT12 was determined in 75 day old male TG9 mice, treated with vehicle or ritonavir since 6 weeks of age, by Western Blot analysis as described in Methods. Western blots are representative data for n = 6 animals/group. Representative full-length blots are presented in Supplementary Fig. 1. Quantitative data are normalized to GAPDH controls and are shown as means ± SEM for n = 6 animals/group *p < 0.01 vs. Veh, ^#^p < 0.05 vs. Veh.

The decrease in PPARα and CD36 expression suggested a decrease in fatty acid oxidation as a source of energy, prompting the question whether there are concomitant changes in myocardial glucose transporters GLUTs −1, −4, and −12. GLUT1 and GLUT12 protein expression increased 50–70% in response to chronic ritonavir treatment (Fig. 7A,C, respectively). Left ventricular GLUT4 protein expression was similar between mice treated with ritonavir or vehicle (Fig. 7B). These data suggest that chronic ritonavir exposure results in compensatory changes in GLUT protein expression to maintain (GLUT4) or increase (GLUT1and GLUT12) expression and support a potential switch from fatty acid oxidation to increased glucose utilization as a primary source of energy.

**Figure 8:**
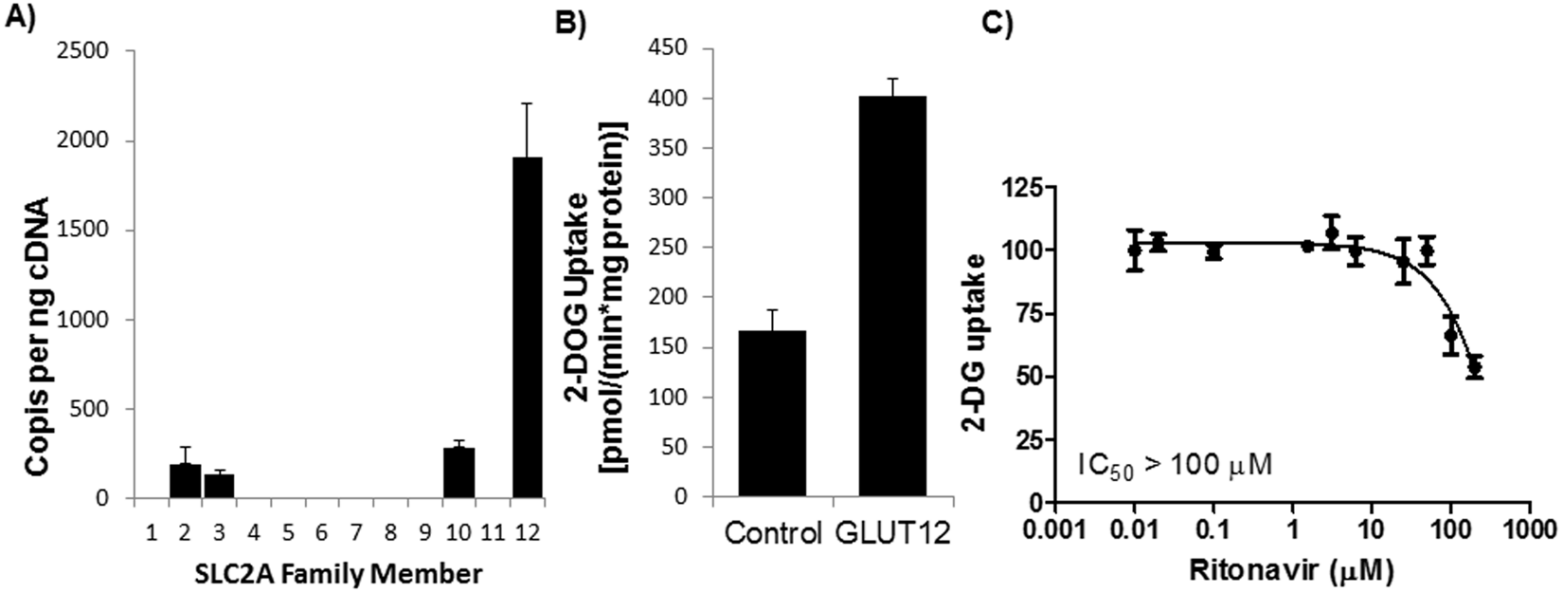
GLUT12 OE Cell Characterization and Effects of Ritonavir on GLUT12 Activity. (**A**) Quantitation of GLUT family members in GLUT12 overexpressing was determined as described in Methods. Glucose transport as determined by 2-DOG uptake was determined in vehicle-treated cells (**B**) or in the presence of increasing concentrations of ritonavir (**C**). Data represent the mean ± S.E. of three independent measurements and in (**C**) were normalized to fit by nonlinear regression analysis using GraphPad Prism software.

Ritonavir inhibits both GLUTs −1 and −4^16^. To determine whether ritonavir inhibits GLUT12, we stably overexpressed human GLUT12 in HEK293 cells and decreased endogenous GLUT1 expression via shRNA knockdown^27^. The levels of other GLUTs were low or undetectable (Fig. 8A). There was a ~2.5-fold increase in glucose transport over control, non-transfected HEK293 cells (Fig. 8B). A dose-response experiment indicates that ritonavir does not specifically inhibit GLUT12-mediated glucose transport, as quantified by 2-deoxyglucose uptake (Fig. 8C).

To investigate whether the beneficial effects of chronic ritonavir treatment observed in TG9 mice are more general, we investigated the effects of chronic ritonavir treatment on cardiac function in the closed-chest ischemia-reperfusion (I/R) model of myocardial injury^28^. C57BL/6 J mice were subjected to 90 min of ischemia followed by 2 weeks of reperfusion while maintained on the vehicle or ritonavir chow admixture (0.1% w/w) for 2 weeks. Transthoracic echocardiography at the end of the 2-week reperfusion period revealed significant improvement of cardiac contractile function in ritonavir-treated animals as compared to vehicle-treated controls (Table 1). Cardiac output was significantly increased as was dV/dt-d, a measure of diastolic relaxation. Ejection fraction and systolic volume showed a trend toward improvement (p = 0.084 and 0.091 respectively). Consistent with the prior study by Gupta *et al*. where ritonavir was found to have cardioprotective against isoproterenol-induced myocardial necrosis^29^, our current results indicate that chronic ritonavir treatment does not impair cardiac function in mice subjected to I/R injury and even improves some parameters.

**Table 1:** Echocardiography results in closed-chest I/R mice treated chronically with ritonavir.

Parameters	Vehicle (n = 10)	Ritonavir (n = 10)	P-value
Heart rate (beats/min)	634 ± 9.5	664 ± 10.7	0.052
EDV (ml)	87 ± 8.9	79 ± 5.9	0.465
ESV (ml)	67 ± 8.8	56 ± 5.3	0.284
EF (%)	24 ± 2.0	30 ± 2.5	0.084
SV	20 ± 1.4	24 ± 1.3	0.091
CO	13 ± 0.8	16 ± 0.8	0.026
dV/dt-s	0.62 ± 0.04	0.69 ± 0.03	0.125
dV/dt-d	0.826 ± 0.04	1.061 ± 0.06	0.004

Transthoracic echocardiographic images were obtained on conscious C57Bl/6 mice at 2 weeks post ischemia/reperfusion as described in Methods. Data are represented as the average ± SEM (n = 10/group). EDV, end-diastolic LV volume; ESV, end-systolic LV volume; EF, ejection fraction [(EDV–ESV)/EDV]; SV, systolic volume; CO, cardiac output; dV/dt-d, peak rate of LV volume increase in diastole; dV/dt-s, peak rate of LV volume decrease in systole.

## Discussion

The observed effects of chronic pharmacologic blockade of GLUT proteins in two distinct murine models of cardiomyopathy provide novel insight into dynamic compensatory changes in glucose transporter biology and the role normal glucose homeostasis in the context of heart failure. In contrast to the glucose intolerance and mortality observed following acute administration of ritonavir to adult TG9 mice with dilated cardiomyopathy, improved glucose homeostasis, insulin sensitivity and survival was observed with chronic drug exposure. This was accompanied by decreased mRNA levels of PPARα and CD36 in LV isolated from mice chronically treated with ritonavir. Studies by Finck *et al*. showed that mice expressing PPARα specifically in the heart (MHC-PPARα) develop cardiomyopathy and have enhanced sensitivity to ischemic insult, effects that are exacerbated when mice are fed a high-fat diet^30^. Cardiac fatty acid uptake was significantly increased in MHC-PPARα mice while reduced expression of genes involved in cardiac glucose utilization was associated with decreased cardiac glucose uptake^31^. In addition, MHC-PPARα mice deficient in CD36 (MHC-PPARα mice crossed to CD36−/−) fed a high fat diet exhibited improved cardiac function, decreased fatty acid uptake and increased GLUT4 expression with concomitant increases in cardiac glucose uptake^32^. These results suggest that PPARα and CD36 play a primary role in a switch from cardiac fatty acid metabolism to glucose substrate utilization that improves cardiac function. While we did not measure cardiac fatty acid uptake in this study, decreased cardiac expression of PPARα and CD36 suggests a switch from fatty acid oxidation to glucose as an alternate fuel source.

In muscle, overexpression of PPARα (MCK-PPARα) causes insulin resistance, glucose intolerance and diabetes in animals fed a high-fat diet^33^. The fatty acid receptor CD36 was increased 5.4-fold in muscle of MCK-PPARα mice, and the expression of GLUTs 1 and 4 fell by half, resulting in increased fatty acid and decreased glucose uptake. We observed similar effects but in the opposite direction in TG9 mice chronically treated with ritonavir: A 70% decrease in PPARα is associated with a 50% decrease in CD36 in soleus muscle. The changes, which were also seen in the heart, suggest reduced fatty acid uptake and oxidation in muscle. Although we did not observe a concomitant reduction in PPAR and CD36 in adipose tissue, we did observe reduction of the insulin resistance markers RBP4 and resistin in this organ. The decrease in heart and skeletal muscle is likely reflective of the predominant role of skeletal muscle in glucose disposal following a glucose challenge. In contrast, adipose tissue accounts for a relatively minor direct role in systemic glucose uptake but functions as an important endocrine organ^34^. The net effect of these integrated changes was significantly improved lifespan, glucose tolerance and insulin sensitivity. Chronic ritonavir treatment may improve some aspects of cardiac function in a model of ischemia-reperfusion injury; it clearly did not exacerbate cardiac dysfunction. The improvements in TG9 mice chronically treated with ritonavir were also associated with increased GLUT1 and −12 expression in LV. Transgenic mice overexpressing GLUT12 have improved glucose tolerance and insulin sensitivity^12^; however, a role of GLUT12 as a basal glucose transporter in the heart rather than an insulin responsive transporter is consistent with the role of glucose transport in cardiac function in the setting of myocardial stress^35^. In addition, a ~4-fold increase in the expression of GLUT12 has been observed in the left ventricle of the pacing-induced canine model of cardiac hypertrophy^11^. The current study suggests a primary role for changes in GLUT protein and fatty acid-related gene expression; however, the observed beneficial effects on cardiac function and systemic glucose homeostasis is likely complex and may involve additional mediators such as changes in inflammatory status, cytokine production and redox status^29,36^.

While additional mechanisms likely participate in these beneficial effects the current study data are consistent with the acute versus chronic heart failure mouse model(s) and supporting clinical evidence. This includes the clinical effects observed in human patients who are exposed to ritonavir. Despite proven success in reducing viral load and prolonging survival, the adverse metabolic effects of PIs continue to pose significant risk to the development of cardiovascular morbidity in the aging HIV infected population worldwide. This concern has been validated in several clinical studies^37,38^. Although the number of patients affected is significant, clinically recognized cardiovascular morbidity is seen in only a small percentage of this patient population^39^. Attempts to reconcile the apparent discrepancy between clinical prevalence of cardiac dysfunction in humans and drug-induced effects in the laboratory setting have included consideration of the limitations of translating data from rodent models to human disease^40^ and drug pharmacokinetics in experimental and treatment conditions^41^. Several other potential confounding factors including the effects of HIV infection itself, associated inflammation, and environmental influences would be predicted to increase rather than decrease cardiac morbidity. While the relatively acute effects of PIs on glucose homeostasis (in both humans and rodents)^18,42^ has aided in identifying the molecular mechanism of these changes, HIV infected patients are typically treated with antiretroviral therapies chronically. Over time, numerous compensatory changes can occur in gene transcription, signal transduction, and metabolite concentrations leading to significant distal effects in target organs. Supporting this hypothesis is the observation of differences in measured changes in insulin sensitivity in acute versus chronic drug exposure experiments^14,18,43–45^. The observation that intermittent PI exposure is associated with increased CV risk is in agreement of the hypothesis that the time of drug exposure may contribute differently to the direct cardiac effects^46^. Although the mechanism of this clinical observation is unknown, the current data suggest that intermittent glucose transport blockade would not allow induction of compensatory changes in the expression of GLUTs −1 and −12.

Taken together these data demonstrate that chronic pharmacologic blockade of glucose transport is linked to modest improvement in cardiac function and lifespan together with improvement in metabolic outcomes. This appears to be due in part to modulation of fatty acid oxidation and glucose utilization pathways. In addition to changes in facilitative glucose transporter expression, it is likely that other molecular mechanisms also participate in the beneficial effects observed and highlights the continued need to explore bidirectional relationships between systemic insulin sensitivity and cardiac function with respect to temporal sequence and magnitude of effect.

## Methods

### Materials

Ritonavir (Norvir) was obtained from Abbott pharmaceuticals (Abbott Park, IL). Ritonavir, in pure drug form and used for *in vitro* studies was provided by the NIH AIDS Reagent Program. GLUT1 and GLUT12 polyclonal antibodies were kind gifts from Mike Mueckler and Kelle Moley, respectively (Washington University, St. Louis, MO). GLUT4 antibody was custom produced by Invitrogen using a peptide corresponding to the 16 carboxyl-terminal residues of GLUT4. Human GLUT12 plasmid was obtained from the DNASU Plasmid Repository (Tempe, AZ). GAPDH monoclonal antibody was purchased from Sigma (St. Louis, MO). Secondary anti-mouse and anti-rabbit antibodies were from LI-COR (Lincoln, NE). Unless noted, all other reagents were purchased from Sigma (St. Louis, MO).

### Animal Care

All mouse studies were approved by the Animal Studies Committee at Washington University School of Medicine and conform to the Guide for the Care and Use of Laboratory Animals published by the National Institute of Health. Mice were housed in the animal facility at Washington University under standard light/dark cycles and fed standard mouse chow diet and water ad libitum. The TG9 dilated cardiomyopathy model was developed by transgenic, high-level cardiac-specific expression of the cre recombinase protein driven by the α-myosin heavy chain promoter, as previously described^26^. The line is maintained in the FVB/N strain background. The characteristic development and progression of dilated cardiomyopathy in this mouse strain has been extensively characterized^24,26^. Starting at 6 weeks of age, male and female TG9 mice were given daily intraperitoneal (i.p.) injections of ritonavir or vehicle control (10% ethanol, 10 mg/kg body weight) until 75 days of age (male) or time of death (female). Female mice were used for lifespan measurements and to prevent undue stress on the animals for this endpoint, males were used for the glucose and insulin tolerance tests, and tissue harvest. Both sexes exhibit the same developmental and phenotypic characteristics of heart failure^24,26^. Harvested tissue was placed immediately in liquid nitrogen and stored at 80 °C pending further analysis. For mouse myocardial ischemia-reperfusion studies, eight-week-old C57BL/6 J male mice were instrumented for subsequent left anterior descending (LAD) coronary artery occlusion as described^47^ and then randomly assigned to either a vehicle (powdered chow) or ritonavir (0.1% (w/w) ritonavir powdered chow admixture) group. After two weeks, animals were subjected to LAD occlusion for 90 minutes, followed by reperfusion and a 2 week recovery period. Animals were maintained on the vehicle or ritonavir chow throughout the study. Cardiac function was determined by echocardiography and then the animals were sacrificed and the left ventricles were dissected out and frozen for RNA and protein analyses.

### Echocardiography

Transthoracic echocardiographic images were obtained on conscious C57Bl/6 mice using a Vevo 2100 Imaging System (VisualSonics, Toronto, Ontario, Canada) equipped with a 30 MHz linear-array transducer at 2 weeks post ischemia/reperfusion. Quantification of left ventricular structure and function by echocardiography was done as described previously^48^.

### RNA isolation and PCR

Total RNA was isolated using the TrizolW Plus RNA Purification System (Invitrogen), and one microgram of RNA was reverse transcribed using qScript cDNA Supermix (Quanta Biosciences). RT-PCR and quantitation were performed in triplicate using Power SYBRW Green PCR Master Mix (Applied Biosystems, Foster City, CA) using the validated primers as described^10^. Mouse primer sequences are as follows: BNP FW- 5′ TCACCGCTGGGAGGTCACTC 3′, BNP RV- 5′ GTGAGGCCTTGGTCCTTCAAG 3′, CD36 FW- 5′ ATTGCGACATGATTAATGGCA 3′, CD36 RV- 5′ GATGGACCTGCAAATGTCAGA 3′, PPARα FW- 5′ CAAGGCCTCAGGGTACCACT 3′, PPARα RV- 5′ TTGCAGCTCCGATCACACTT 3′, RBP4 FW- 5′ ACATGGTGGGCACTTTCACAG 3′, RBP4 RV- 5′ CCAAGTTTGG AATCCCAAGCC 3′, Resistin FW- 5′ TGTTGTGAATTTTACTACTTGTCC 3′, Resistin RV- 5′ TGCACACGTGGTACTCAAGTG 3′ and Actin FW- 5′ GATTACTGCTCTGGCTCCTAG 3′, Actin RV- 5′ GACTCATCGTACTCCTGCTTG 3′. To quantify GLUT mRNA transcript levels in the GLUT12 OE cells, qPCR was performed with standard curves generated using plasmids containing each human GLUT (DNASU)^27^.

### Western Blot Analysis

Left ventricular myocardium was harvested from mice immediately following euthanasia and flash frozen in liquid nitrogen. Lysates were prepared by homogenizing the frozen ventricles in lysis buffer containing 1% Triton X-100, 0.5% NP-40, 300 mM NaCl, 20 mM Tris, pH 7.5, 2 mM EDTA, 2 mM EGTA, 1 mM sodium vanadate, 50 mM sodium fluoride, 10 mM sodium pyrophosphate, and protease inhibitor cocktail in PBS. Lysates were incubated on ice for 30 minutes and cleared by centrifugation at 15000 × g for 10 minutes at 4 °C. Protein concentration was determined using the Pierce BCA Protein Assay Kit (Pierce Biotechnology, Rockford, IL). Western blot analysis was then performed using 30 μg of total protein per lane. GLUT1, GLUT4, and GLUT12 antibodies recognizing the COOH-terminus of the transporters were used at a 1:1000 dilution in 5% milk in TBS-T. GAPDH was used as an internal control for loading variability. Blots were imaged and relative protein levels were determined using the Odyssey Infrared Imaging System Version 3.0 (LI-COR Biosciences, Lincoln, NE).

### Glucose and Insulin Tolerance Tests

Following a 5 h fast, fasting glucose level was sampled from tail vein (time zero); the mice received a solution of 50% dextrose (2 g/kg) via gavage (glucose tolerance test) or 0.2U/kg (0.02U/ml) insulin via intraperitoneal injection (insulin tolerance test). Approximately 5 ul of blood was sampled from tail veins at regular intervals over the following 2 h. Blood glucose was immediately determined using an Acenscia Contour glucometer (Bayer Health care LLC, Tarrytown, NY). OGTTs were measured at 8 and 10 weeks of age (2 and 4 weeks treatment, respectively), and ITT was measured at 9 weeks of age (3 weeks of treatment- to let the mice recover between OGTT measurements).

#### GLUT12 overexpressing (GLUT12 OE) cell line generation

HEK293 cells were stably transfected with human GLUT12 in the pcDNA 3.1(-) hygro plasmid (Life Technologies) as described previously^27^. Single clones were selected by comparing their abilities to transport radiolabeled glucose. To reduce background, native hGLUT1 was knocked down using shRNA as described^27^.

#### Measurements of radiolabeled glucose uptake

Uptake of [^3^H]2-deoxy-D-glucose (2-DOG) into GLUT12 OE cells was measured in HEPES buffered saline at room temperature for 4 min as described previously^27^. Briefly, tissue culture plates were pretreated with 25 μg/ml polyethyleneimine (Fluka, catalogue number P3143) in 150 mM NaCl for 20 min to let cells adhere. GLUT12 OE cells were plated at 400,000 cells/ml overnight. Cells were then washed with glucose-free HEPES buffer and starved for 30 min. The uptake of 2-DOG (50 μM) was measured in glucose-free HEPES buffer for 4 min at 37 °C. Non-specific uptake was measured in non-transfected HEK293 cells containing the shRNA GLUT1 knockdown and was subtracted from the experimental values. Data are plotted as specific activity or percent uptake relative to unexposed HEK293 cultures. Data were normalized to fit by nonlinear regression analysis using GraphPad Prism software.

#### Statistical analyses

The data are reported as means ± SEM. Differences between control and experimental values were determined by two-tailed t-test, with significance determined at the P < 0.05 level.

## Electronic supplementary material

Supplemental Information
