# Supplementary material for: Metabolic and Cardiac Adaptation to Chronic Pharmacologic Blockade of Facilitative Glucose Transport in Murine Dilated Cardiomyopathy and Myocardial Ischemia
Source: Sci Rep. 2018 Apr 24;8:6475. doi: 10.1038/s41598-018-24867-1 (PMC5915485; doi:10.1038/s41598-018-24867-1)
Supplement: Supplementary file 1 — Supplemental Information [file 41598_2018_24867_MOESM1_ESM.pdf]

Metabolic and Cardiac Adaptation to Chronic Pharmacologic Blockade of Facilitative Glucose Transport in Murine Dilated Cardiomyopathy and Myocardial Ischemia

Monique R. Heitmeier[1], Maria A. Payne[1], Carla Weinheimer[2], Atilla Kovacs[2], Richard C. Hresko[1], Patrick Y. Jay[1,3], and Paul W. Hruz[1, 4]

[1]Department of Pediatrics, [2]Department of Internal Medicine, [3]Department of Genetics, [4]Department of Cell Biology and Physiology, Washington University School of Medicine

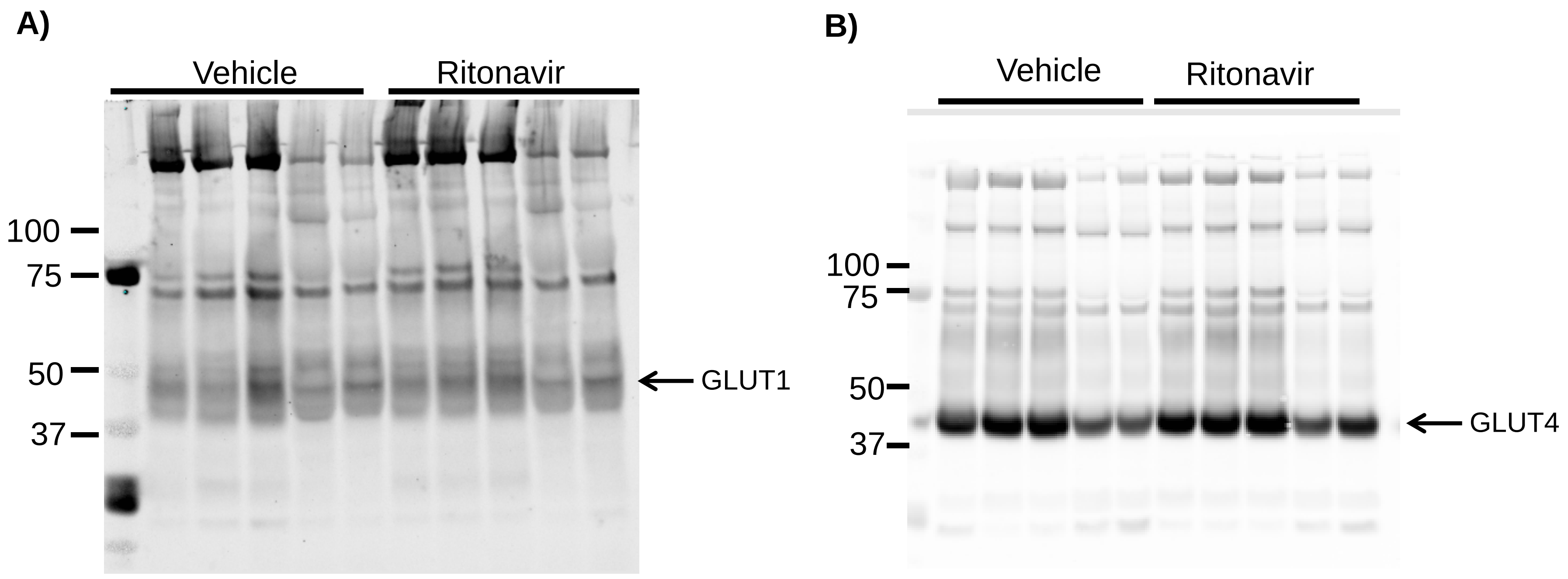

Supplementary Figure1: Full-length blots for Figure 6. Effects of Chronic Ritonavir Treatment on GLUT Protein Expression by Skeletal Muscle. Protein expression of A) GLUT1, B) GLUT4 was determined in 75 day old male TG9 mice treated with vehicle or ritonavir since 6 weeks of age by Western Blot analysis as described in Methods.

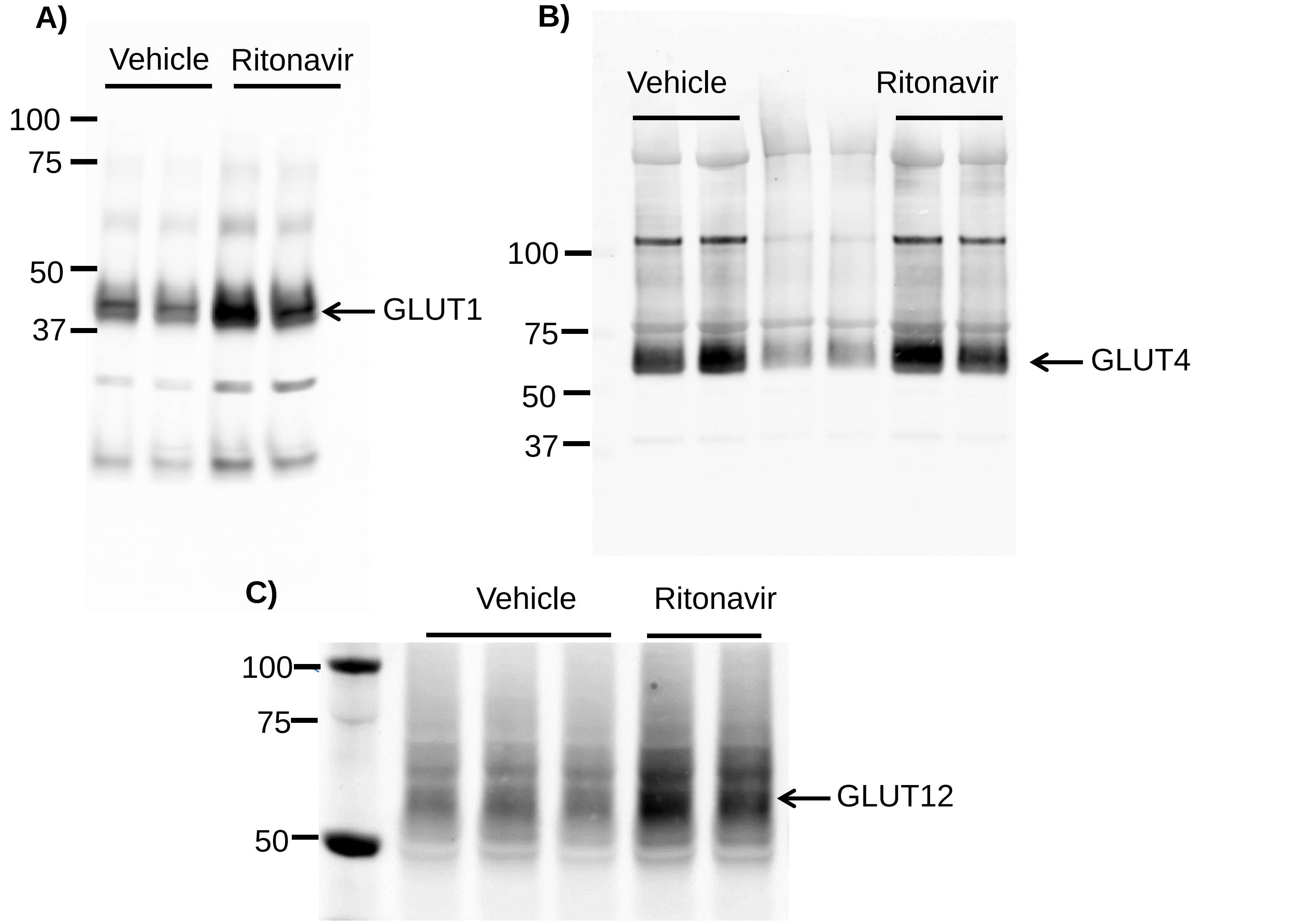

Supplementary Figure 2: Full-length blots for Figure 7. Effects of Chronic Ritonavir Treatment on GLUT Protein Expression by Left Ventricle. Protein expression of A) GLUT1, B) GLUT4, and C) GLUT12 was determined in 75 day old male TG9 mice, treated with vehicle or ritonavir since 6 weeks of age by Western Blot analysis as described in Methods.
